# Supplementary material for: Time‑resolved multi-omic analysis of paclitaxel exposure in human iPSC‑derived sensory neurons unveils mechanisms of chemotherapy‑induced peripheral neuropathy
Source: Cell Death Dis. 2026 Feb 10;17(1):211. doi: 10.1038/s41419-026-08445-2 (PMC12921266; doi:10.1038/s41419-026-08445-2)
Supplement: Supplementary file 15 — Dataset 1. [file 41419_2026_8445_MOESM15_ESM.zip › CDD_Open_Data/04_RNA_Seq/Timeline_panel_plots_etc_2023-01-23_1609.zip!/Panel_Plots_etc/Evidence_Plots/6h/evidence_plot_standard contrasts_msigdb_go_bp_6h_PTX_vs_DMSO_ID2_M29111.pdf]

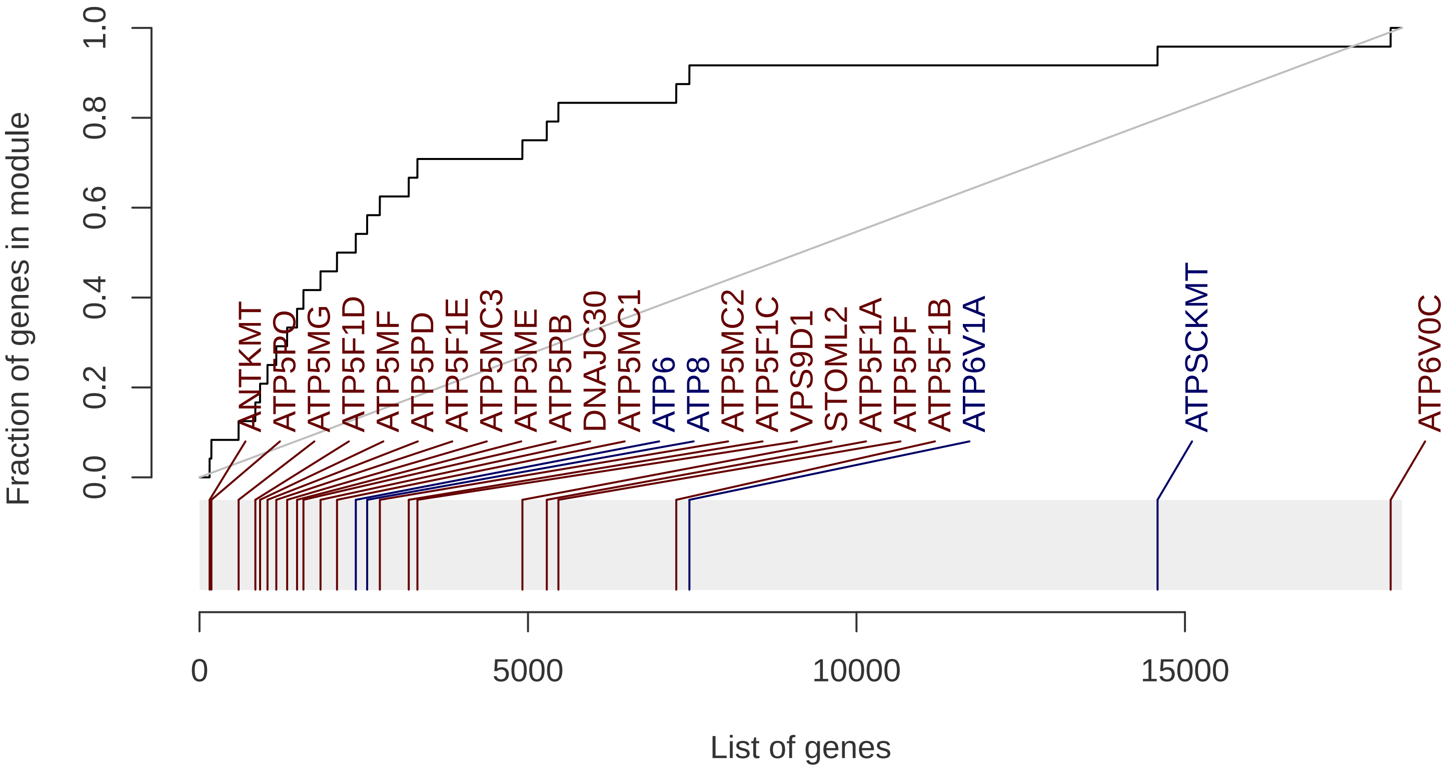
Fraction of genes in module
0.0
0.2
0.4
0.6
0.8
1.0
ANTKMT
ATP5PO
ATP5MG
ATP5F1D
ATP5MF
ATP5PD
ATP5F1E
ATP5MC3
ATP5ME
ATP5PB
DNAJC30
ATP5MC1
ATP6
ATP8
ATP5MC2
ATP5F1C
VPS9D1
STOML2
ATP5F1A
ATP5PF
ATP5F1B
ATP6V1A
ATPSCKMT
ATP6V0C
0
5000
10000
15000
List of genes
